# Tobacco addiction in the psychiatric population and in the general population ^1^

**DOI:** 10.1590/1518-8345.2202.2945

**Published:** 2017-12-04

**Authors:** Renata Marques de Oliveira, Jair Lício Ferreira Santos, Antonia Regina Ferreira Furegato

**Affiliations:** 2 PhD, RN, Hospital das Clínicas de Marília (HC-Famema), Marília, SP, Brazil.; 3 PhD, Full Professor, Faculdade de Medicina de Ribeirão Preto, Universidade de São Paulo, Ribeirão Preto, SP, Brazil.; 4 PhD, Full Professor, Escola de Enfermagem de Ribeirão Preto, Universidade de São Paulo, PAHO/WHO Collaborating Centre for Nursing Research Development, Ribeirão Preto, SP, Brazil.

**Keywords:** Tobacco Use, Tobacco Use Disorder, Epidemiology, Cross-Sectional Studies, Mental Health, Psychiatric Nursing

## Abstract

**Objective:**

To estimate the degree of tobacco addiction and identify independently associated factors by comparing the psychiatric population of secondary and tertiary care with the general population of the primary healthcare network.

**Method:**

This is a cross-sectional epidemiological study, conducted in a municipality of São Paulo, with 134 smokers of a Mental Health Outpatient Unit (MHOU), a Psychiatric Hospital (PH), and a Primary Healthcare Unit (PHU). Data were collected by means of individual interviews, recorded on a mobile device. Data were statistically processed using Stata/12

**Results:**

Of the 134 participants, 54.5% were women. While 49.1% of the psychiatric population (MHOU/PH) had medium/high nicotine addiction, 58.3% of smokers of the general population had very low/low dependency. The Poisson regression model indicated a higher prevalence of smokers with high dependence among men (PR = 1.41), people aged 49 years or less (15 - 29 years, PR = 4.06, 30 - 39 PR = 2.96 years, 40 - 49 years PR = 1.84), with severe mental disorders (PR = 3.05), with anxiety disorders/other (PR = 3.98), and with high suicide risk (PR = 1.55).

**Conclusion:**

Nicotine dependence was greater in the psychiatric population than in the general population. The independent factors associated with severe dependence were sex, age group, diagnosis, and current risk of suicide. These results trigger reflection among nurses on the need to focus more attention on a neglected subject in mental health services.

## Introduction

Mental disorders can cause intense anguish, reduce quality of life, self-esteem, and social and work performance, and affect the interpersonal relationships and family ties of sufferers^1^ ^-^ ^3^.

In addition to the emotional and social losses, psychiatric patients suffer from impaired health, a lower life expectancy, and a greater occurrence of somatic complications. People with severe and persistent mental disorders live an estimated 25 years less than the general population, and smoking is one of the main causes of reduced life expectancy^4^ ^-^ ^6^.

Although 22% of the US population suffers from a mental disorder, these patients consume half of the cigarettes produced in the country and represent 46% of tobacco-related deaths, which reveals the severity of the epidemic. Moreover, the highest levels of nicotine dependence and the lowest levels of smoking cessation are found among people with severe mental disorders^7^ ^-^ ^12^.

High tobacco consumption has a significant impact on the financial life of people with mental disorders. These smokers can spend up to 30% of their monthly income on cigarettes, whereas more than 4% is considered prejudicial. Difficulties in obtaining cigarettes can lead people with mental disorders to engage in demeaning practices such as stealing cigarettes and smoking cigarette butts, which reduce their self-esteem and self-respect^13^ ^-^ ^15^.

Furthermore, mental disorders can be more severe among smokers. Patients who smoke suffer more outbreaks (delusions and hallucinations), thoughts of suicide or suicide attempts, and psychiatric hospitalizations^(7, 11, 16-18)^.

 In light of scientific evidence of the damage smoking causes to the psychiatric population, tobacco use in this population cannot continue being overlooked by nursing professionals, as, according to the Law of Professional Practice (COFEN Resolution 311/2007), nursing is committed to promoting the health of people as a whole.

This study addresses the following questions: 1) Do the levels of tobacco dependence differ between the psychiatric population and the general population of the primary health network? 2) Which factors are independently associated with tobacco addiction in these two populations?

Although the use of tobacco by the psychiatric population has been the subject of national and international scientific literature in recent years, this is the first Brazilian study to compare tobacco addiction between the psychiatric population and the general population.

The aim of this paper was to estimate the degree of nicotine addiction and identify independently associated factors by comparing the psychiatric population of secondary and tertiary care with the general population of the primary healthcare network.

## Method

This is a cross-sectional epidemiological study conducted in a municipality in the interior of the state of São Paulo, Brazil. 

To compare tobacco dependence between the psychiatric population and the general population, this study was conducted with three groups from different health services of the municipality: Mental Health Outpatient Unit (MHOU), Psychiatric Hospital (PH), and Primary Health Unit (PHU).

The sample was composed of 134 smokers: 34 of the MHOU, 76 of the PH, and 24 of the PHU. The number of participants was not the same in all three services because this study is part of a larger project to identify different aspects of smoking in the psychiatric population and in the general population. The sample was calculated using the total number of participants per unit, including smokers, ex-smokers, and non-smokers. 

The sample calculation for the larger project (significance level (α) = 5%, beta (β) = 10%, estimated smokers in PH = 60%, estimated smokers in the MHOU = 40%) indicated the need to interview 126 persons per unit (total sample = 378). The sample of this study is smaller since it only included smokers.

The inclusion criteria were 1) self-declared smoker; 2) residing in the municipality; 3) attending the health service in the period of data collection. The exclusion criteria were 1) under 15 years of age; 2) diagnosis of mental retardation; 3) problematic use of alcohol or illegal substances without psychiatric comorbidities; 4) difficulty communicating verbally. 

The project was recorded in Plataforma Brasil/CONEP No. CAAE 21101113.3.0000.5393 and approved by the Research Ethics Committee of the Escola de Enfermagem de Ribeirão Preto - EERP/USP (308/2013). The technical teams of the study sites were consulted regarding the possibility of collecting data. 

The participants signed two copies of an informed consent statement, one for the participant and one for the researcher. Whenever a subject was unable to consent participation, the guardian also signed the statement. 

Three patients under the age of 18 signed the consent form and their guardians signed the informed consent statement to authorize the participation of the minors in this research.

Data were collected using the following three instruments:

1) Questionnaire for subject identification, prepared by the researchers especially for this project, according to the following variables: sex (female, male); age group (15 - 29 years, 30 - 39 years, 40 - 49 years, 50 - 59 years, ≥ 60 years); schooling (illiterate, elementary, secondary, higher), marital status (single, married, separated/divorced, widowed); psychiatric diagnosis (severe mental disorders, anxiety disorders/other, without diagnosis); current use of antipsychotics (first generation, second generation, first and second generation, does not apply); alcohol use (uses, used, never used); illicit substance use (uses, used, never used); importance of tobacco (below average, above average).

The variable importance of tobacco was obtained on a scale of 0 to 10, according to the importance the smokers’ attributed to tobacco. In the descriptive analysis, the average importance of tobacco was 6.6. This value was used to create two categories: below average and above average.

2) Scale for monitoring current suicide risk: consisting of six questions to classify the current risk of suicide as low, moderate, or high. The subjects should answer the five questions according to the events of the previous month: 1) Did you think you would be better off dead or did you wish you were dead? 2) Did you want to harm yourself? 3) Did you think of suicide? 4) Did you think of ways to commit suicide? 5) Did you attempt suicide? The last question addressed the subject’s entire life: 6) Have you ever attempted suicide?^19^
3) Fagerström Test for Nicotine Dependence (FTND): This test comprises six questions that investigate cigarette smoking patterns (first cigarette of the day, difficulty to refrain from smoking in non-smoking locations, most satisfying cigarette of the day, number of cigarettes, period smoking is more frequent, and whether subject smokes even when sick). Each response has a score, and the sum of these scores determines the level of tobacco dependence, as follows: very low (0 to 2 points); low (3 to 4 points); medium (5 points); high (6 to 7 points); and very high (8 to 10 points). The test was validated for use in Brazil (test retest 0.915 and Cronbach’s alpha 0.642) and it is considered the “standard” test to assess nicotine dependence ^(^ ^20^.

Data were collected through individual interviews in a reserved room. They were conducted by a single interviewer. The answers of the participants were recorded on a mobile device (tablet) using the application TabacoQuest, especially designed for this project^21^.

The responses of the subjects, marked by the researcher in the application, were automatically transferred to Excel spreadsheet and subsequently transferred to Stata (version 12) for statistical processing.

We used descriptive statistics tools to characterize the participants (average, standard deviation, minimum, maximum, and absolute and relative frequency). The variable tobacco dependence was subjected to bivariate analysis by calculating the Prevalence Ratio (PR) and its respective confidence interval (CI 95%).

Multivariate analysis was performed using Poisson regression, with degree of tobacco addiction (FTND score: ≤ 5 and ≥ 6) as the outcome. The model was adjusted with time of tobacco use as the exposure control (offset variable).

It was possible to dichotomize the FTND scores since this cutoff point is recognized in scientific literature^9^. 

We selected the independent variables with p< 0.20 in bivariate analysis and those considered relevant in scientific literature. The criterion of not exceeding the limit of 10 cases by variable was also observed^22^. Thus, the model could have up to 13 variables (134/10 = 13.4).

The independent variables included in the model were sex; age group; antipsychotics currently in use; main psychiatric diagnosis; current risk of suicide; alcohol; illegal substances; and importance of tobacco use. The variance inflation factors (VIF) were calculated to assess the presence of collinearity.They all had VIF<10. The average VIF was 3.

The results were discussed according to scientific literature.

## Results

Most of the 134 participants were single women who had finished elementary school. The average age of the participants was 46 years (15 to 78 years, SD = 14). 

Approximately three-quarters of the participants were diagnosed with severe mental disorders (schizophrenia, schizoaffective disorder, mood or personality disorder), and a significant portion used first generation antipsychotics. 

About a third of the subjects had a high risk of suicide. Most participants stated they were consuming alcohol, and a minority admitted using illegal substances (Table 1).

**Table 1:** Absolute and relative frequency (%) of the participant characterization variables (n = 134) - Marília (SP), Brazil, 2014

**Variables**	**n**	**%**
**Location**		
MHOU*	**34**	**25.4**
**PH†**	**76**	**56.7**
**PHU** ^**‡**^	**24**	**17.9**
**Sex**		
**Female**	**73**	**54.5**
**Age group (years)**		
**15 to 29**	**22**	**16.4**
**30 to 39**	**25**	**18.7**
**40 to 49**	**29**	**21.6**
**50 to 59**	**35**	**26.1**
**≥ 60**	**23**	**17.2**
**Education**		
**Illiterate**	**7**	**5.2**
**Elementary**	**86**	**64.2**
**Secondary**	**32**	**23.9**
**Higher**	**9**	**6.7**
**Marital status**		
**Single**	**74**	**55.2**
**Married**	**29**	**21.6**
**Separated/Divorced**	**20**	**14.9**
**Widowed**	**11**	**8.2**
**Main psychiatric diagnosis**		
**No diagnosis**	**16**	**11.9**
**Severe mental disorders**	**98**	**73.1**
**Anxiety disorders/other**	**20**	**14.9**
**Current use of antipsychotics**		
**Not applicable**	**40**	**29.8**
**1** ^**st**^ **generation**	**56**	**41.8**
**2** ^**nd**^ **generation**	**19**	**14.2**
**1** ^**st**^ **and 2** ^**nd**^ **generation**	**19**	**14.2**
**Current risk of suicide**		
**No risk**	**56**	**41.8**
**Low**	**25**	**18.7**
**Moderate**	**10**	**7.5**
**High**	**43**	**32.1**
**Alcohol**		
**Never used**	**19**	**14.2**
**Uses**	**75**	**56.0**
**Used**	**40**	**29.8**
**Illicit substances**		
**Never used**	**89**	**66.4**
**Uses**	**17**	**12.7**
**Used**	**28**	**20.9**
**Total**	**134**	**100.0**

*MHOU: Mental Health Outpatient Clinic †HP: Psychiatric Hospital ‡PHU: Primary Health Unit

Seventy-one smokers (53%) considered that tobacco had above average importance.

The 134 smokers had started smoking, on average, 28.5 years ago. A shorter smoking time was found among the PH smokers (MHOU = 31, PH = 25, PHU = 35).

**Figure 1:**
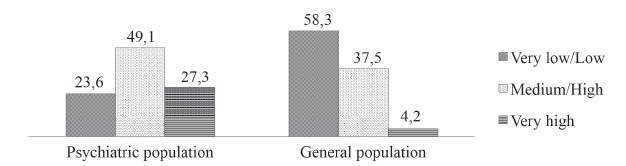
Relative frequency (%) of tobacco addiction among the psychiatric population of secondary and tertiary care compared to the general population of the primary health system - Marília (SP), Brazil, 2014

Of the 134 smokers, 29.9% were classified with a very low/low dependence on tobacco, 47% with medium/high dependence, and 23.1% with very high dependence. When comparing the two populations, approximately half of the psychiatric population (MHOU and PH) had medium/high level of dependency, while most of the smokers of the general population attended at the primary health unit had very low or low tobacco addiction (Figure 1).

**Figure 2:**
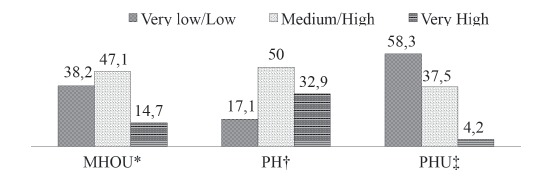
Relative frequency (%) of degree of tobacco dependence, according to the study site (MHOU: Mental Health Outpatient Unit; PH: Psychiatric Hospital; PHU: Primary Health Unit) - Marília (SP), Brazil, 2014

In terms of the study location, a similar prevalence of smokers with medium/high degrees of dependence was observed at the MHOU and the PH. The highest frequency of participants with a very high degree of dependence was found at the PH. At the PHU, the number of smokers classified as very high was negligible (Figure 2).

*MHOU: Mental Health Outpatient Clinic †HP: Psychiatric Hospital ‡PHU: Primary Health Unit

The bivariate analysis provided statistical evidence that high tobacco addiction (FTND score ≥ 6) is linked to serious mental disorders, the use of second generation antipsychotics, the concomitant use of first and second generation antipsychotics, the high risk of suicide, and the current and past use and illicit substances. 

The Poisson regression model indicated a greater prevalence of highly dependent smokers among men, people aged 49 years or under, and people with severe mental disorders, anxiety disorders/other, and high suicide risk. 

In the model, the association of tobacco addiction with antipsychotics and with illicit substances was no longer detected, suggesting that this association occurred due to the interference of other variables (Table 2).

**Table 2:** Crude and adjusted Prevalence Ratio (PR) for degree of tobacco dependence according to FTND - Marília (SP), Brazil, 2014

**Variables**	**Tobacco dependence** FTND*		**Crude**	**Adjusted** ^**†**^
	**≤ 5 points** **n (%)**	**≥ 6 points** **n (%)**	**PR (CI 95%)** ^**‡**^	**PR (CI 95%)**
**Sex**				
**Female**	**34 (46.6)**	**39 (53.4)**	**1**	**1**
**Male**	**21 (34.4)**	**40 (65.6)**	**1.23 (0.93. 1.63)**	**1.41 (1.01. 1.95)** ^**§**^
**Age group (years)**				
**≥ 60**	**11 (47.8)**	**12 (52.2)**	**1**	**1**
**15 to 29**	**8 (36.4)**	**14 (63.6)**	**1.22 (0.74. 2.02)**	**4.06 (1.86. 8.87)** ^**§**^
**30 to 39**	**6 (24)**	**19 (76)**	**1.46 (0.93. 2.28)**	**2.96 (1.64. 5.32)** ^**§**^
**40 to 49**	**10 (34.5)**	**19 (65.5)**	**1.26 (0.78. 2.01)**	**1.84 (1.05. 3.25)** ^**§**^
**50 to 59**	**20 (57.1)**	**15 (42.9)**	**0.82 (0.47. 1.42)**	**0.90 (0.50. 1.60)**
**Diagnosis**				
**No diagnosis**	**13 (81.3)**	**3 (18.7)**	**1**	**1**
**Severe mental disorders**	**33 (33.7)**	**65 (66.3)**	**3.54 (1.26. 9.90)** ^**§**^	**3.05 (1.06. 8.80)** ^**§**^
**Anxiety disorders/other**	**9 (45)**	**11 (55)**	**2.93 (0.98. 8.76)**	**3.98 (1.40. 11.33)** ^**§**^
**Current use of antipsychotics**				
**Not applicable**	**21 (52.5)**	**19 (47.5)**	**1**	**1**
**1** ^**st**^ **generation**	**25 (44.6)**	**31 (55.4)**	**1.17 (0.78. 1.74)**	**0.77 (0.52. 1.14)**
**2** ^**nd**^ **generation**	**4 (21)**	**15 (79)**	**1.66 (1.11. 2.48)‡**	**1.21 (0.79. 1.83)**
**1** ^**st**^ **and 2** ^**nd**^ **generation**	**5 (26.3)**	**14 (73.7)**	**1.55 (1.02. 2.37)‡**	**0.93 (0.57. 1.52)**
**Current risk of suicide**				
**No risk**	**30 (53.6)**	**26 (46.4)**	**1**	**1**
**Low**	**12 (48)**	**13 (52)**	**1.12 (0.70. 1.79)**	**1.14 (0.71. 1.81)**
**Moderate**	**4 (40)**	**6 (60)**	**1.29 (0.72. 2.30)**	**1.22 (0.70. 2.12)**
**High**	**9 (20.9)**	**34 (79.1)**	**1.70 (1.23. 2.35)** ^**§**^	**1.55 (1.02. 2.35)** ^**§**^
**Alcohol**				
**Never used**	**11 (57.9)**	**8 (42.1)**	**1**	**1**
**Uses**	**19 (47.5)**	**21 (52.5)**	**1.25 (0.68. 2.28)**	**0.84 (0.45. 1.59)**
**Used**	**25 (33.3)**	**50 (66.7)**	**1.58 (0.91. 2.74)**	**1.01 (0.58. 1.76)**
**Illicit substances**				
**Never used**	**45 (50.6)**	**44 (49.4)**	**1**	**1**
**Uses**	**2 (11.8)**	**15 (88.2)**	**1.78 (1.36. 2.34)** ^**§**^	**1.46 (0.95. 2.23)**
**Used**	**8 (28.6)**	**20 (71.4)**	**1.44 (1.06. 1.98)** ^**§**^	**1.19 (0.76. 1.88)**
**Importance of tobacco**				
**Below average**	**31 (49.2)**	**32 (50.8)**	**1**	**1**
**Above average**	**24 (33.8)**	**47 (66.2)**	**1.30 (0.97. 1.75)**	**1.12 (0.77. 1.63)**
**Total**	**55 (41)**	**79 (59)**		

*FTND: Fagerström Test for Nicotine Dependence; †Adjusted: Poisson Multiple Regression Model; ‡PR (CI 95%): Prevalence Ratio and Confidence Interval of 95%; § evidence of statistical association (p< 0.05)

The multiple regression model revealed that the prevalence of smokers with high dependence was 41% higher among men than among women. 

**Figure 3:**
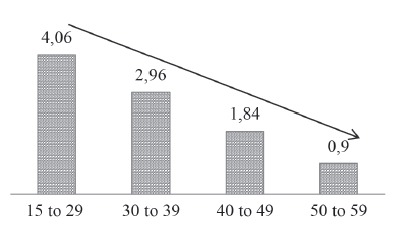
Adjusted Prevalence Ratio for high degree of tobacco dependence, according to age group (reference group: elderly (≥ 60 years)) - Marília (SP), Brazil, 2014

The participants between 15 and 29 years old showed a prevalence of smokers with a degree of dependence that was 4.06 times greater than among the elderly participants. The prevalence ratio dropped as the aged increased, suggesting a dose-response effect (Figure 3).

Regardless of the variables included in the Poisson regression model, the prevalence of smokers with an FTND score ≥ 6 was, respectively, 205% and 298% greater among participants with severe mental disorders and among participants with anxiety disorders/other disorders compared to those with no diagnosis. 

The participants considered at high risk for suicide had a 1.55 greater prevalence of being smokers with high nicotine dependence than those who did not present risk of suicide.

The data obtained from this sample suggest that the importance smokers attribute to tobacco is not related to the intensity of their addiction. In the bivariate model, this relationship may have appeared with a larger sample since the lower limit of the confidence interval was close to 1 (a limit greater than 1 would have shown evidence of an association). In the multiple model, however, the prevalence ratio dropped and the CI limits (95%) clearly showed that there was no possibility of association.

## Discussion

Tobacco addiction was more intense in the psychiatric populations, especially among patients at the psychiatric hospital.

The high dependency on tobacco of this population is consistent with national and international findings^(8-11, 23)^. 

In the general population, assisted in the primary care system, there was a predominance of smokers with very low/low tobacco addiction. A recent national survey indicated that 81% percent of smokers of the general Brazilian population showed low dependence on tobacco^24^. 

The multivariate analysis revealed a greater dependence among men, younger age groups, people with severe mental disorders, anxiety disorders/other disorders, and people at high risk of suicide.

The higher prevalence of smokers with tobacco addiction among men is a reflection of the ideals promoted by the tobacco industry. In advertisements, smoking is associated, among other things, with the image of masculinity/virility. Although, years later, the tobacco industry started to include women in their target audience, smoking among women did not escalate due to social pressure^25^ ^-^ ^26^.

In addition to the influence of the industry and social acceptance, the greater dependence of men in relation to women is attributed to the female hormones that protect women against more intense dependence on tobacco^27^ ^-^ ^28^. 

This fact is important because it indicates that strategies to treat tobacco dependence should be different for men and women. Men should respond better to medication that targets addiction while women should respond better to treatment oriented toward the behavioral aspects of smoking^27^ ^-^ ^28^. 

This insight is valuable for nurses since, as members of a healthcare team and a multidisciplinary setting, they must actively use their technical and scientific knowledge to plan the best strategies to help patients stop smoking.

Greater tobacco addiction among the young population, in comparison to the elderly, was maintained even after adjusting sex, psychiatric diagnosis, current use of antipsychotics, risk of suicide, alcohol use, illicit substance use, and importance attributed to tobacco. 

This result causes concern since young people are less prone to seek psychiatric treatment. For anti-smoking interventions to reach this population, nurses and other health workers must create effective strategies for individuals who have not yet been included in the mental health network^29^.

The strong association between tobacco addiction and mental disorders was expected because of the high prevalence of smoking in psychiatric population. The greater dependence on tobacco of this population can be related to the increased need for cigarettes to relieve psychiatric symptoms.

A cohort study conducted in the USA (n=43.093) revealed that each psychiatric diagnosis, in addition to the main psychiatric diagnosis, increases the probability of classifying the individual as a heavy smoker by 67% (≥ 24 cigarettes/day)^29^. This effect, however, could not be assessed in this study because it only contains the main diagnoses. 

A Brazilian study conducted with patients with mental disorders admitted at Psychiatric Unit of General Hospitals revealed that 78% of smokers used cigarettes as a way of self-medicating psychiatric symptoms. In the study, 79% of the patients stated smoking relieved anxiety, 57.3% stated smoking improved their mood, and 29.2% said smoking increased their concentration. Regardless, some patients acknowledged that relief of these symptoms was temporary, leading them to smoke more frequently^(10, 30)^.

The association between intense tobacco addiction and the high risk of suicide was maintained in the multiple model. 

The relationship between suicide and smoking was highlighted in cohort studies that identified tobacco use and high nicotine dependence as a risk factor for suicidal behavior, even after adjusting for psychiatric variables. In terms of the dose-response, evidence shows that the greater the number of cigarettes consumed in a day, the higher the risk of suicide. There is also evidence that smoking cessation reduces the risk of suicide^31^ ^-^ ^40^.

Some authors sought to explain the association between tobacco use and suicide with the use of neurotransmitters, and found that decreasing the activity of the enzyme monoaminaoxidase (MAO-A and MAO-B) can increase impulsive behavior, which is one of the predisposing factors for suicide attempts^41^ ^-^ ^43^. 

More extensive research and social and therapeutic actions that address this issue are recommended to reduce the risk of suicide^32^.

A study conducted in the US detected a reduction in the risk of suicide after tobacco control actions, such as increasing tax on cigarettes and smoking-free locations. It was estimated that an increase of one dollar in taxes on cigarettes could reduce the risk of suicide by 10.5%. Considering the absolute number of suicides in 2012, this percentage corresponds to a reduction of 4,000 suicides per year in the US^44^.

The association between tobacco addiction and suicide risk is an important finding for nursing, as, despite the Brazilian law (12.546/2011) that prohibits smoking in collective environments, smoking is still found in many Brazilian mental health services. Considering nursing staff must closely observe patients with risk of suicide, especially during psychiatric hospitalization, allowing psychiatric patients to smoke goes against the ethical principle of non-malfeasance. 

In this study, the association of tobacco addiction with the use of alcohol and illicit substances was not consistent and characterized the degree of dependence as dichotomous (≤ 5 points and ≥ 6 points), although these cutoff points are recognized in scientific literature^9^.

The results of this study can support the actions of nurses since they confirm a higher prevalence of smokers with intense tobacco dependence among the psychiatric population and identify, by means of a multiple model, the variables that are independently associated with addiction. The resulting knowledge and debate on smoking can help include smoking in the dialogue with patients and the multidisciplinary team.

One of the limitations of this study is the adopted method (cross-sectional study), which does not allow causal inference.

## Conclusion

Tobacco addiction was higher in the psychiatric population at secondary and tertiary care levels than in the general population that uses the primary health network.

In the multiple model, the resulting independent factors associated with severe dependence were sex, age group, diagnosis, and current risk of suicide.

These results should encourage Brazilian nursing professionals to pay closer attention to a subject that is neglected in mental health services.
